# Consistent Condom Use and Dual Protection Among Female Sex Workers: Surveillance Findings from a Large-Scale, Community-Based Combination HIV Prevention Program in Tanzania

**DOI:** 10.1007/s10461-019-02642-1

**Published:** 2019-08-23

**Authors:** Gaspar Mbita, Amasha Mwanamsangu, Marya Plotkin, Caterina Casalini, Amani Shao, Gissenge Lija, Dorica Boyee, Angella Ramadhan, Neema Makyayo, Ramadhani Mlange, Raymond Bandio, Megan Christofeld, Albert Komba

**Affiliations:** 1 Jhpiego Tanzania, Dar es Salaam, Tanzania; 2 Jhpiego, Baltimore, MD USA; 3 National Institute for Medical Research (NIMR), Mwanza-Center, Mwanza, Tanzania; 4 Ministry of Health, Community Development, Gender, The Elderly and Children, Dodoma, Tanzania; 5 Engender Health, Dar es Salaam, Tanzania

**Keywords:** HIV, Female sex workers, Family planning, Contraception, Dual method use, HIV prevention, Sexually transmitted infection, Condom use, Tanzania

## Abstract

In Tanzania, HIV infection remains much higher among female sex workers (FSWs) than among other adult women. In addition to HIV, sexually transmitted infections (STIs) and pregnancy prevention are major concerns for FSWs in Tanzania. This study used a programmatic surveillance approach to examine protection against STIs/HIV and unintended pregnancy (dual method use) among FSWs in an outreach-based HIV prevention, care, and treatment program in Tanzania. 119,728 FSWs made a first visit to services served by the Sauti Project from January 2016 to September 2017. Of these 79,774 were current contraceptive users—of those, 4548 (5.7%) took a contraceptive as well as condoms, the study measure of dual family planning (FP) method use. Ninety-one percent (n = 4139) of FSWs taking dual FP methods were provided with an injectable in addition to condoms. Dual method use was lower in this study than in research studies in the region, highlighting potential differences between findings from research studies and evidence from a routine service provision setting. Self-reported consistent condom use among FSWs was 16.1%. The findings call for further research and programs to address FSW agency to increase dual protection against STIs/HIV and unintended pregnancy.

## Introduction

New HIV infections in east and southern Africa declined 29% between 2010 and 2016 [1]. However, HIV incidence among certain high-risk populations, such as female sex workers (FSWs), remains high in comparison to the general population [2]. According to global estimates, FSWs were average 13.5 times more likely to be living with HIV than women in the general population in 2011, and HIV prevalence among FSWs in sub-Saharan Africa averaged 36.9% [2]. In Tanzania, while HIV prevalence has declined among women aged 15–49 (from 7.7% in 2003 to 6.2% in 2017) [3, 4], a 2013 behavioral and biological surveillance survey in Tanzania found an estimated HIV prevalence among FSWs of 26.6% [5].

Use of a family planning (FP) method can be considered a proxy for access to reproductive health care for this vulnerable population. FP use is important for preventing unintended pregnancies, which are common among FSWs [6]; use of a barrier method also reduces STI/HIV acquisition and transmission. A study of FSWs in three sub-Saharan African countries (Burkina Faso, Swaziland, and Togo) showed significant and consistent unmet need for FP [7]. Although new biomedical breakthroughs such as pre-exposure prophylaxis have the potential to prevent HIV infection, they are not yet widely available. Therefore, male and female condoms remain an essential tool for preventing HIV infection and other STIs among high-risk populations.

Consistent condom use is an important method of protection against both pregnancy and STIs. Multiple studies have found that consistent condom use among FSWs is low in sub-Saharan Africa: 23.5% in Swaziland [8], 40% in the Democratic Republic of the Congo [9], and from 21% consistent use with steady partners to 40% consistent use with new clients in Iringa region, Tanzania [10].

Both individual- and environmental-level factors influence variations in consistent condom use among FSWs. Stigma, shaming, and reluctance of health care providers to offer health services to FSWs have been reported in multiple settings from sub-Saharan Africa to India [11, 12]. Although targeted services for FSWs have been shown to effectively increase access to and use of services in India, no increase was noted for FSWs in cities in South Africa, Mozambique, and Kenya [13]. The literature documents several individual-level factors that predict consistent condom use: power to negotiate condom use (self-efficacy) [14, 15], knowledge that condom use prevents HIV infection [16], having been engaged as a commercial sex worker for a shorter amount of time, and having been tested for HIV [9].

Given the reported inconsistent use of condoms among FSWs, we look at provision of dual methods—that is, the receipt of any FP method or combination of methods that protects from both pregnancy and STIs (including HIV). In this study, dual protection was measured as FSWs who receive an FP method, or report using a method, in addition to taking condoms at a clinical service visit in the context of a HIV prevention program.

All studies examining dual method use in sub-Saharan Africa report low usage, even among FSWs who are at high risk of STIs, HIV, and unintended pregnancy. Multiple studies among women in the general population have described low dual method use as well. A household survey in South Africa found that 6.3% of adult women used dual FP methods at last sex [17]; another study found that only 7% of adolescent girls reported using dual FP methods [18]. In Botswana, dual method use was described as 3.5% among women aged 15–49 from a population survey [19]. Only 8% of FSWs in Swaziland reported dual method use in the last 30 days [8]; however, 38% of FSWs in Kenya reported consistent dual method use [20].

The Sauti Project is a US President’s Emergency Plan for AIDS Relief (PEPFAR)-funded, United States Agency for International Development (USAID)-administered, community-based HIV prevention program that provides comprehensive HIV prevention, treatment, and reproductive health services to key and vulnerable populations in Tanzania. Sauti was scaled up from five to 14 of Tanzania’s 25 regions between 2015 and 2018. Since its inception, Sauti has reached more than 1 million individuals from key and vulnerable populations in Tanzania, including almost 120,000 FSWs (Sauti project summary of annual results, Oct 1, 2017–Sept 30, 2018). Sauti provides services through mobile clinical outreach teams, which reach brothels or other transmission “hot spots” such as nightclubs, guest houses, and truck drivers’ parking places. Clinical services provided include HIV prevention, testing, care, and treatment services; FP; and screening and counseling for gender-based violence, tuberculosis (TB), STIs, alcohol, and drugs. Sauti services are designed to be stigma-free, with health care providers trained to work with populations that experience barriers to accessing reproductive health services.

The current study uses a program surveillance design, drawing on program monitoring data from the Sauti Project’s clinical records. The findings provide insights into real-world service delivery in the context of a comprehensive, outreach-based HIV prevention and treatment program. The study’s definition of dual method use differs from that used in many other studies as it is based on actual provision of contraception methods and condoms in a service delivery setting. This study reports on FSWs’ choice of FP methods when they have access to FP services. Given the scale of the Sauti Project, the study also presents findings from a larger population of FSWs in Tanzania than has previously been described.

The following analytical questions underpin the study:

1. What are the demographic and HIV risk characteristics of FSWs served through the Sauti Project?
2. What proportion of FSW reported consistent condom use and what were the correlates of use?
3. What proportion of FSW reported dual method use and what were the correlates of dual method use?

The results presented here will be useful to program planners and policymakers, especially those planning HIV prevention and reproductive health services for FSWs in sub-Saharan Africa.

## Methods

### Program Surveillance Design

Surveillance is a descriptive measurement design in which a population is monitored for a disease or condition in order to plan control or prevention activities [21]. Whether active or passive, the methodology is intentionally designed to contribute to disease control or prevention activities among the population. Surveillance has also been defined as “watchfulness over a community” [21]. The current study is best described as programmatic surveillance. Although public health surveillance programs typically rely on public health authorized records, such as birth or death registration, surveillance is also possible in the programmatic context [22]. In this study, the population being observed is FSWs receiving services through the Sauti Project. The Sauti Project database provides information on these and other Sauti Project beneficiaries, and is regularly analyzed to provide information to program planners to improve services. This fills the essential function of the surveillance feedback loop for improving health status of the population [22].

### Program Description and Geographic Locations

**Fig. 1:**
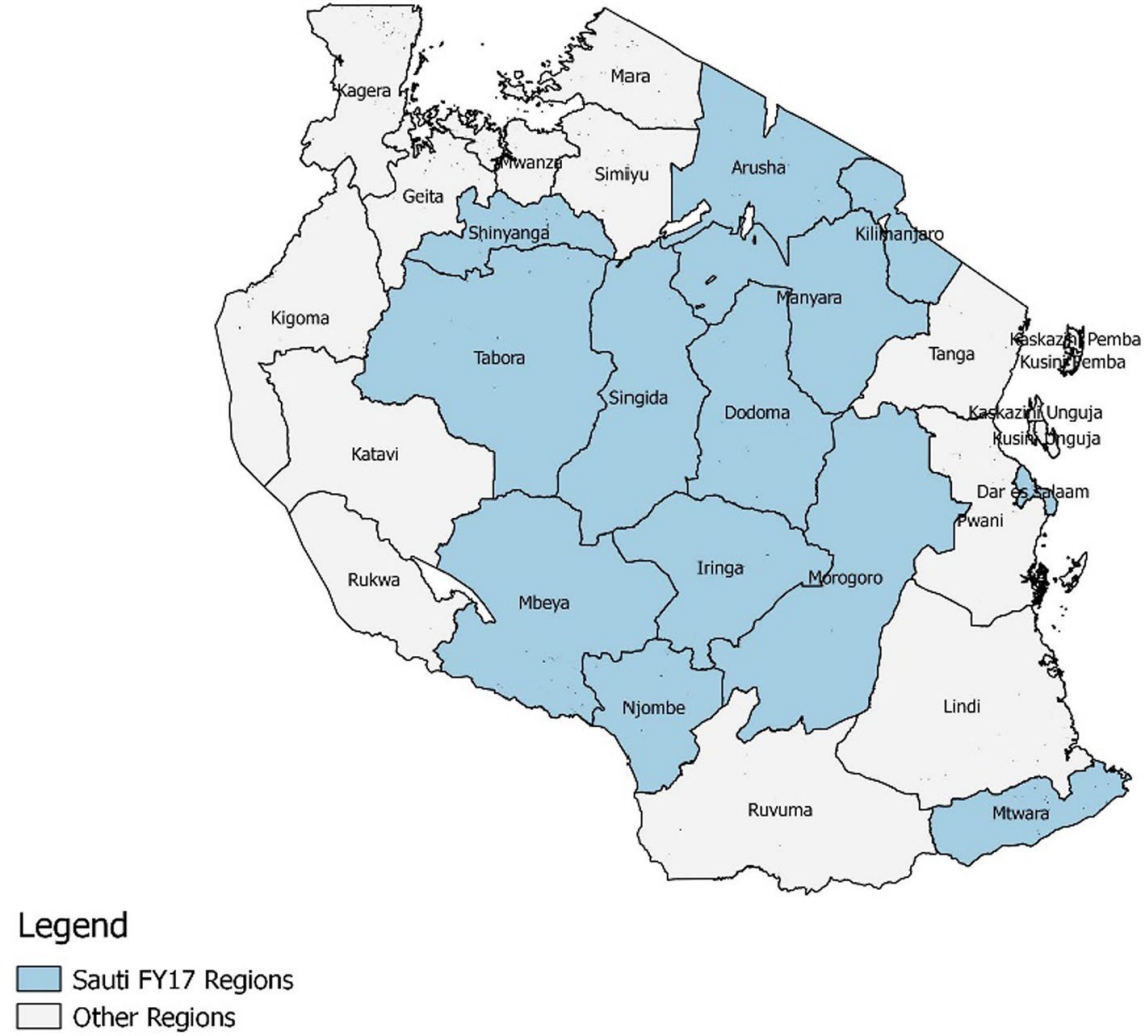
Map of Sauti Project regions included in analysis

This paper presents information on FSWs served by the Sauti Project from January 2016 to September 2017. We selected this time period because of the availability of cleaned data as well as use of a similar intake form. Data from this period were subject to data quality reviews that indicated the records were 80% complete. During this time, the Sauti Project operated in 14 regions of Tanzania (Arusha, Dodoma, Kilimanjaro, Morogoro, Iringa, Njombe, Mbeya, Singida, Dar es Salaam, Mtwara, Shinyanga, Tabora, Manyara, and Songwe) (see Fig. 1), providing its services to beneficiaries free of charge.

The Sauti program model is to use regionally based mobile clinical teams (trained nurses and clinicians) to provide biomedical services to vulnerable and high-risk populations at hot spots, which are defined as areas of high HIV transmission. Examples of hotspots include brothels, mining and fishing villages, plantations, truck drivers’ parking places, and social venues such as bars, nightclubs, and guesthouses. Services are provided under a tent or in rented rooms from Monday to Saturday, day and night.

Clinical services offered include sexual risk assessment; syndromic screening for STIs; screening for TB, alcohol and drug use, and gender-based violence; HIV testing; escorted linkage to HIV care and treatment; and referral to social, legal, and medical care. FP services include provision of female and male condoms, injectables, oral contraceptive pills, and implants (offered at outreach services); and intrauterine contraceptive device (IUCD), vasectomy, or tubal ligation (offered via escorted referral to a government health facility).

### Study Population

For this analysis, an FSW is defined as a female Sauti beneficiary who reports exchanging sex for cash or goods as her primary and current source of income (over half of her monthly income). Only first-visit FSW aged 18–49 were included in the analysis. The majority of Sauti beneficiaries only have one service visit due to the mobile nature of services and health seeking behaviors of Sauti beneficiaries. Because multiple visits are rare, longitudinal analysis of the Sauti database is rare. To avoid, double counting, we included first visit Sauti beneficiaries only. All first visits of women attending Sauti services from January 2016 to September 2017 who self-reported the characteristics meeting our definition of an FSW were included in the analysis.

### Condom Use and Dual FP Method Use Definitions

Condom use was taken from self-reports of the women attending Sauti services, using a clinical intake form completed by a health care provider. In this analysis, consistent condom use was defined as a response of “always” to the question, “Was a condom (male or female) used with permanent or regular partners or paying partners in the last 1 month?” Responses of “sometimes” and “never” were defined as inconsistent condom use.

The definition of dual method use in this analysis differs from definitions in many of the research study results appearing in the literature. Because we describe a routine service delivery setting, our definition centers around service provision. Other studies use a time-bound definition of dual method use, for example, “ever used dual protection” [23], “dual protection at last sex act” [17–19], and “use of dual protection in the last 30 days” [8]. We have defined dual method use as being provided condoms in addition to either starting or reporting continuing a FP method during the Sauti visit.

### Data Collection Tools

The data in the Sauti database comes from a standardized Ministry of Health, Community Development, Gender, the Elderly and Children (MOHCDGEC)-approved client record called the Health Screening and Service Tool (HSST), version 8.

### Data and Data Management

We drew data from Sauti’s program database, which holds de-identified individual-level records of Sauti beneficiaries. Health care providers enter client data into paper-based client records during the clinical consultation. Dedicated data entry staff, supervised by regional monitoring and evaluation officers, enter data from the filled forms (minus identifying information) into the Sauti database. The Jhpiego Tanzania office has an informatics and data management team, which supports the integrity of data in the Sauti database. Validation rules are in place for many variables in the database to prevent data entry errors, and data quality assessments are conducted regularly.

### Ethical Oversight

Secondary data review permission was obtained by the institutional review boards of Johns Hopkins Bloomberg School of Public Health (IRB No 00006673) and the National Institute of Medical Research of Tanzania (*NIMR/HQ/R.8c/Vol IX/678*).

### Analysis

The variables for the analysis were determined through a consensus among experts in HIV prevention, care, and treatment and FP in Tanzania. The main outcomes for this analysis were consistent condom use and dual method use. Consistent condom use was coded “1” if a respondent indicated that she always used a condom with permanent, regular or paying partners in the last month, and “0” if a respondent indicated sometimes or never using a condom with those partners in the last month. We defined dual method use as the beneficiary being provided with condoms, in addition to either starting or reporting continuing a FP method. This variable was coded as “1” if a woman was provided condom in addition to any FP method and as “0” if she was not provided with condoms. Age was collected as a continuous variable generated by deducting date of birth from stated age at visit. Afterwards, we stratified age into four categories (18–24, 25–34, 35–44 and 45–49 years). Marital status was grouped into three categories by combining married and cohabiting together also combining divorced and widowed together. Sexual partners (reported as casual, paying and non-paying/regular partners) were self-reported as the number of sexual partners in the past 3 months. The phrasing of the question made it impossible to analyze by type of sexual partner. The information on number of partner was stratified into three groups: 1–2 partners, 3–4 partners, 5 or more partners. We generated a variable on syndromic STIs by combining information on reported STI symptoms (urethral discharge, genital ulcer, genital sore, genital vesicle, swollen lymph nodes and ano-rectal discharge). We coded this variable as “1” if a “yes” was reported as having any of the symptoms.

All statistical analyses were performed using Stata 14 (College Station, Texas, 77845, USA). We used Pearson’s Chi square test for categorical variables to examine associations between sociodemographic characteristics, condom use, and dual method use; and multivariable logistic regression to determine factors associated with dual method use. Bivariate analysis was conducted on sociodemographic characteristics, and those significantly associated with consistent condom use and dual method use at p < 0.10 were entered into multivariable models. In multivariable regression analyses, we adjusted for confounding factors including age, HIV status, marital status, number of sexual partners, and ever been pregnant. We considered results with a *p* value of < 0.05 to be significant. We were not able to control for parity since that question is not present on the clinical intake form. Less than 5% of FSW included in the analysis were missing age, marital status and STIs information. We ran a sensitivity analysis to ascertain that there were no statistical differences in outcomes variables (consistent condom use and dual method use) between participants with missing information and those with information.

## Results

### Demographics and Risk Factors of FSWs in the Sauti Project

From January 2016 to September 2017, 119,468 FSWs received Sauti services for the first time. Half (51.5%) of these FSWs were aged 25–34, 44.3% were single, virtually all (91.8%) reported having ever been pregnant, and 7.9% tested positive for HIV at their first Sauti visit (women self-reporting known HIV-positive status were not tested) (Table 1). Of the FSWs seen for a first visit, 16.1% self-reported consistent condom use (Table 1) and 5.7% had dual method use (that is, both being provided with condoms and being provided with another FP method/reporting using another FP method) (Table 3).

**Table 1:** Social behavioral characteristics of female sex workers receiving Sauti clinical services for the first time (N = 119,468)

Characteristics	n	%
Age (years)		
18–24	29,757	24.9
25–34	61,534	51.5
35–44	23,847	20.0
45–49	4330	3.6
Marital status		
Single	52,897	44.3
Married/cohabiting	50,240	42.1
Divorced/widowed	16,331	13.6
Type of partner (currently reported) ^α^		
Regular and paying	72,282	60.5
Paying only	47,186	39.5
Number of reported sexual partners (including paying and regular/nonpaying) currently reported		
1–2	39,050	32.7
3–4	44,726	37.4
5 +	35,692	29.9
Ever been pregnant		
No	9790	8.2
Yes	109,678	91.8
HIV test result at Sauti visit		
Negative	106,627	89.3
Positive	9404	7.9
Not tested	3437	2.8
Syndromic STI screening		
Negative	2478	2.1
Positive	14,985	12.5
Not screened	102,006	85.4
Consistent condom use within last month		
Consistent condom use	19,317	16.1
Inconsistent condom use	100,151	83.9
Family planning method taken/reported during clinical visit		
Not using	39,694	33.2
Pills	19,549	16.4
Condoms alone	37,947	31.8
Intrauterine device	460	0.4
Implants	4556	3.8
Injectable	12,714	10.6
Other	4548	3.8

^a^Number of paying sex partners of FSW had in the last week as reported by FSW at the initial visit of Sauti service delivery

### Consistent Condom Use

Higher proportions of consistent condom use (with all partners, including paying and non-paying) were reported among FSWs aged 18–24 (17.1%), single FSWs (18.8%), FSWs with more than five sexual partners (15.8%), FSWs who had never been pregnant (12.1%), and FSWs who tested positive for HIV (10.0%) (Table 2). A multivariable analysis showed that FSWs aged 25–34 years were 1.4 times (95% CI 1.4–1.5) more likely to use a condom consistently than FSWs aged 18–24 years. FSWs who reported being single were 1.4 times (95% CI 1.4–1.5) and divorced/widowed were 1.5 times (95% CI 1.5–1.6) more likely to use condoms consistently than FSWs who reported being married/cohabiting. The likelihood of consistent condom use went up steadily with the number of partners reported. FSWs having three or four sexual partners per month were 2.8 times (95% CI 2.6–2.9) more likely and those having five or more partners were 5.2 times (95% CI 4.9–5.5) more likely to use condoms consistently than FSWs with one or two partners. FSWs who reported never being pregnant were 1.5 times (95% CI 1.4–1.5) more likely to use condoms consistently than those who reported ever being pregnant. FSWs who tested positive for HIV were less likely to use condoms consistently (0.6 [95% CI 0.6–0.7]) compared to those who tested negative for HIV.

**Table 2:** Factors associated with consistent condom use among female sex workers

Characteristics	Total N	FSW clients reporting consistent condom use		Adjusted analysis	
		n	%	aOR [95% CI]	*p*-value
Overall	119,468	19,324	16.1		
Age (years)					
18–24	29,757	5102	17.1	Reference	
25–34	61,534	9932	16.1	1.4 [1.4–1.5]	<0.001
35–44	23,847	3738	15.6	1.0 [1.0–1.1]	0.517
45–49	4330	552	12.7	0.8 [0.7–0.9]	<0.001
Marital status					
Single	52,897	9965	18.8	1.4 [1.4–1.5]	<0.001
Married/cohabiting	50,240	6315	12.6	Reference	
Divorced/widowed	16,331	3044	18.6	1.5 [1.5–1.6]	<0.001
Number of reported sexual partners (including paying and regular/nonpaying) currently reported^a^					
1–2	39,050	537	1.4	Reference	
3–4	44,726	7072	15.8	2.8 [2.6–2.9]	<0.001
5 +	35,692	1509	4.2	5.2 [4.9–5.5]	<0.001
Ever been pregnant					
No	9790	1189	12.1	1.5 [1.4–1.5]	<0.001
Yes	109,678	7790	7.1	Reference	
HIV test result at Sauti visit					
Negative	106,627	2871	2.7	Reference	
Positive	9404	942	10.0	0.6 [0.6–0.7]	<0.001
Not tested	3437	499	14.5	0.9 [0.8–1.0]	0.005

N = 119,468

Consistent condom use defined as responses of “always” to the question: did you consistently use a condom with regular and paying partners within last 1 month?

^a^Number of paying sex partners of FSW had in the last week as reported by FSW at the initial visit of Sauti service delivery

### Family Planning Use

**Fig. 2:**
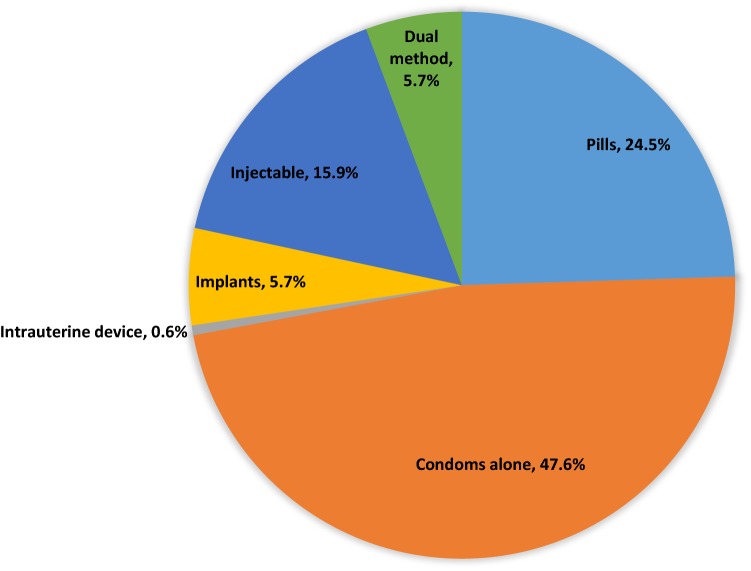
Method mix among female sex workers who use a family planning method (n = 79,774). ^a^*Note* family planning users are defined as those who accepted or continued a contraception method during their Sauti clinical service visit

FP use (FP method taken at the Sauti visit or self-reported continuing use) was 66.8% (n = 79,774). One-third (33.2%) reported not using a FP method and did not take a FP method at their visit. Condoms alone were the most frequently taken FP method (31.8% of all FSWs), followed by pills (16.4%), injectables (10.6%), and implants (3.8%) (Table 1). In terms of method mix, close to half (47.6%, n = 37,947) of FP users took condoms alone at their service visit (Fig. 2).

### Dual Method Use

**Fig. 3:**
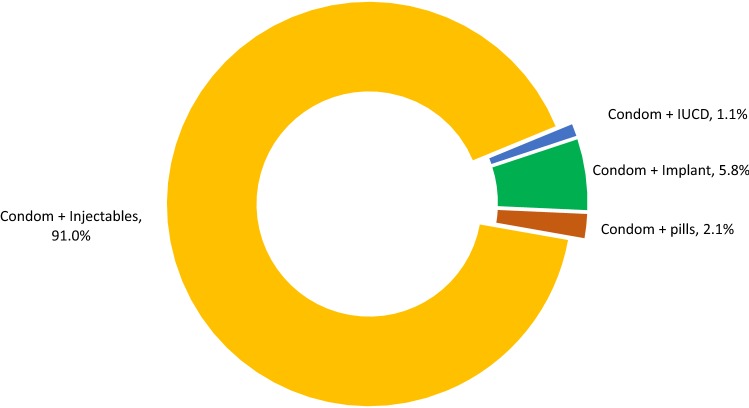
Method mix among dual family planning method users (n = 4548). ^a^*Note* dual method use is defined as taking condoms as well as starting or continuing another contraception method, including pills, injectables, IUCD, or tubal ligation

Out of 79,774 FSWs who were currently using an FP method, 4548 (5.7%) took dual methods, meaning they accepted condoms in addition to starting or continuing another FP method during their Sauti visit (Table 3). The vast majority of FSWs who were using dual FP methods were using condoms and injectables (n = 4139, 91.0%). A small proportion of dual method users used condoms and an implant (5.8%), condoms and pills (2.1%), and condoms and an IUCD (1.1%) (Fig. 3).

**Table 3:** Socio-behavioral characteristics associated with dual method use among female sex workers who are currently using contraception at Sauti clinical services visit

Characteristics	Total (N)	Dual method users n (%)	aOR [95% CI]	*p*-value
Overall	79,774	4548 (5.7)		
Age (years)				
18–24	19,419	927 (4.8)	Reference	
25–34	41,989	2621 (6.2)	1.1 [1.0–1.2]	0.212
35–44	15,622	882 (5.7)	1.1 [1.0–1.2]	0.163
45–49	2744	118 (4.3)	0.6 [0.5–0.7]	< 0.001
Marital status				
Single	35,961	2146 (6.0)	1.0 [1.0–1.1]	0.701
Married/cohabiting	32,743	1717 (5.2)	Reference	
Divorced/widowed	11,070	685 (6.2)	1.1 [1.0–1.2]	0.217
Number of reported sexual partners (including paying and regular/nonpaying) currently reported^a^				
1–2	30,600	1375 (4.5)	Reference	
3–4	26,836	1548 (5.8)	1.5 [1.2–1.7]	< 0.001
5 +	22,338	1625 (7.3)	2.1 [1.8–2.3]	< 0.001
Ever been pregnant				
No	3526	26 (0.7)	Reference	
Yes	68,263	3801 (5.5)	5.4 [3.7–8.0]	< 0.001
HIV status				
Negative	72,607	4103 (5.6)	Reference	
Positive	7167	445 (6.2)	1.1 [1.0–1.3]	0.032
Any syndromic STI				
Negative	1709	50 (2.9)	Reference	
Positive	11,236	1648 (14.7)	5.2 [3.9–7.0]	< 0.001
Not screened	58,844	2129 (3.6)	1.8 [1.4–2.5]	< 0.001

N = 79,774

^a^Number of paying sex partners of FSW had in the last week as reported by FSW at the initial visit of Sauti service delivery

In multivariable analysis, women who screened positive for an STI using syndromic management were 5.2 (95% CI 3.9–7.0) times more likely to take dual methods. Women were more likely to take dual methods if they had ever been pregnant (5.4 [95% CI 3.7–8.0] times more likely compared to those who had never been pregnant); tested positive for HIV during the Sauti visit (1.1 [95% CI 1.0–1.3]) or had more sexual partners (3–4 partners, 1.5 [95% CI 1.2–1.7]; 5 + partners, 2.1 [95% CI 1.8–2.3) (Table 3).

## Discussion

This study presents a new way to look at uptake of dual methods, by presenting findings drawn from actual service provision in a large-scale, comprehensive outreach-based HIV prevention, reproductive health, and care and treatment program. The findings suggest very low levels of uptake of dual methods (condoms plus another contraception method) (5.7%). Self-reports of FSWs also indicate low levels of consistent condom use (16.1%). Taken together, this implies a low level of dual protection (protection against both pregnancy and STI/HIV infection) among this key population.

Uptake of dual methods among Sauti FSWs was low, and generally lower than reported from more purely research-oriented studies. Dual method use among FSWs has been estimated to be 18.0% in Zambia [24], 38.0% in Kenya [20], and 8.0% in Swaziland [8]. Our findings also show that one-third (33.2%) of FSWs reported using no FP method and took no FP method at their Sauti visit, including condoms. Self-reported consistent condom use (with all partners, paying and non-paying) (16.1%) was also lower than in studies conducted in the region: a Swaziland study reported 23.5% consistent condom use [8], a Democratic Republic of the Congo study reported 40.0% [9], and a Swaziland, Togo, and Burkina Faso study showed 17.0% [7]. We acknowledge that the differences may be a byproduct of the methodologies associated with measurement design in a research setting, compared to a service delivery setting. Notably, however, the difference may represent actual differences between what FSWs say they do, what they would like to do, and what they actually do in regard to dual protection when being offered FP and STI protection. We are concerned about the low levels of use of these important protection measures among a highly vulnerable population group in a setting where access to services is not limited. In Tanzania and similar settings, there is a need to emphasize programs that implement interventions aimed at addressing multiple obstacles for family planning uptake concurrently. Such interventions may include community distribution of contraceptive methods, public information campaigns, improved training for health professionals and community health workers, and strengthening of the health infrastructure [25]. Furthermore, we recommend follow-up studies to examine the reasons behind usage patterns so that they can be addressed by appropriate service delivery models.

FSW choices regarding FP use, including dual method use, are complex since they stem from both fertility desires as well as the need to protect against STI infection. Literature indicates that fertility intentions of FSW are an important driver of reproductive health decision-making, including uptake of FP. A study of FSW in southwestern Tanzania indicates that among FSW, who had a median of two children, desire to become pregnant was related to gaining respect in society as well as other strong motivators. The study also revealed a complex pattern of paternity, with 36% of pregnancies among FSW being attributed to non-paying partners such as husbands or boyfriends, 23% to regular clients and 28% to casual clients [26]. In Togo, Burkina Faso, and Swaziland, 19% of FSWs in the study reported that they were trying to conceive [7]. The complexities of decision-making around FP choices and fertility intentions were beyond the scope of the study. The Sauti health care providers providing services may have discussed fertility intentions when providing the service, but this information is not recorded in the HSST tool and thus not available to systematically assess in the study population. However, the findings lead us to recommend that fertility intentions be more comprehensively explored and documented in programmatic monitoring for FSW.

There is limited information on consistent condom use and dual method use in the service delivery setting in sub-Saharan Africa, with no comparable study to which we could contrast our findings. This may be due to described difficulties in publishing descriptive programmatic data rather than experimental study data [22]. A condom use program in India which promoted socially marketed condoms using peer networks was evaluated using two rounds of cross-sectional measurements after 3 years of intervention, with an outcome measure of consistent condom use and syphilis infection [27]. In terms of comparability, not only was the outcome measure very different, the evaluation methodology (behavioral survey) was substantially different to our analysis of routine program data. We would like to see more examples of programmatic evaluation, and recommend further studies or rigorous program evaluation based on service delivery.

### Limitations

This study, conducted in the context of a comprehensive, outreach-based service delivery program, was not designed to systematically measure differences in consistent condom use and dual method use as is done in research studies. However, the number of FSWs in the Sauti Project allowed us to run these analyses with confidence in our findings. Due to the design of the HSST intake form, we were not able to stratify our analysis by paying and non-paying sexual partners of FSW, which is a notable limitation. We will suggest that future iterations of the form allow better design to allow for these analyses. These findings do not represent the nearly 3 years of Sauti Project service delivery. The time period under analysis is the period for which data were clean and changes to the data collection form did not affect comparability of the variables in the analysis. A program as large as Sauti Project, which manages hundreds of thousands of records, experiences some data challenges such as missing data. However, the data used in this analysis was selected because we had confidence in its completeness and quality due to extensive data cleaning and quality assessment. It also be noted that the classification of FSW (derive over half of their income from sex work) was our own definition—some of the women described in this paper might not consider themselves FSWs. There is no information in the literature relevant to the Tanzanian context as to whether there might be a difference in risk behaviors based on a woman’s self-identification of a sex worker or not. Unfortunately, Sauti program data does not allow us to investigate this potential difference in behaviors. This may warrant further investigation.

## Conclusion

This study, the first to look at self-reported consistent condom use and uptake of dual methods among FSWs in a large-scale community program in Tanzania, found low levels of consistent condom use and extremely low uptake of dual FP methods. These findings are concerning, both from the perspective of this high-risk group protecting themselves, and for preventing ongoing transmission of HIV. Notably, these findings occur within a clinical setting—one that is tailored to reach high-risk beneficiaries. If we take away the barrier of availability of access to dual FP methods, utilization was still surprisingly low. Our findings suggest the need to increase awareness and agency among FSWs to increase their use of FP methods that can protect them against both exposure to HIV and unintended pregnancy. Integration of assessment of fertility intentions of FSW into service delivery and into programmatic monitoring may add greater understanding of high quality services.
